# Bat Influenza A(HL18NL11) Virus in Fruit Bats, Brazil

**DOI:** 10.3201/eid2502.181246

**Published:** 2019-02

**Authors:** Angélica Cristine Almeida Campos, Luiz Gustavo Bentim Góes, Andres Moreira-Soto, Cristiano de Carvalho, Guilherme Ambar, Anna-Lena Sander, Carlo Fischer, Adriana Ruckert da Rosa, Debora Cardoso de Oliveira, Ana Paula G. Kataoka, Wagner André Pedro, Luzia Fátima A. Martorelli, Luzia Helena Queiroz, Ariovaldo P. Cruz-Neto, Edison Luiz Durigon, Jan Felix Drexler

**Affiliations:** Charité-Universitätsmedizin Berlin, corporate member of Freie Universität Berlin, Humboldt-Universität zu Berlin, and Berlin Institute of Health, Institute of Virology, Berlin, Germany (A.C.A. Campos, L.G.B. Góes, A. Moreira-Soto, A.-L. Sander, C. Fischer, J.F. Drexler);; Universidade de São Paulo-USP, Instituto de Ciências Biomédicas-ICB, São Paulo, Brazil (A.C.A. Campos, L.G.B. Góes, E.L. Durigon);; Universidade Estadual Paulista Faculdade de Medicina Veterinária de Araçatuba, Araçatuba, Brazil (C. de Carvalho, W.A. Pedro, L.H. Queiroz);; Universidade Estadual Paulista, Instituto de Biociências, Rio Claro, Brazil (G. Ambar, A.P. Cruz-Neto);; Centro de Controle de Zoonoses, São Paulo (A.R. da Rosa, D.C. de Oliveira, L.F.A. Martorelli, A.P.G. Kataoka);; German Centre for Infection Research, Germany (J.F. Drexler);; Martsinovsky Institute of Medical Parasitology, Tropical and Vector-Borne Diseases, Sechenov University, Moscow, Russia (J.F. Drexler)

**Keywords:** influenza A virus, bats, HL18NL11, Atlantic forest, viruses, influenza, zoonoses, Brazil

## Abstract

Screening of 533 bats for influenza A viruses showed subtype HL18NL11 in intestines of 2 great fruit-eating bats (*Artibeus lituratus*). High concentrations suggested fecal shedding. Genomic characterizations revealed conservation of viral genes across different host species, countries, and sampling years, suggesting a conserved cellular receptor and wide-ranging occurrence of bat influenza A viruses.

Influenza A viruses are major causes of human disease and are predominantly maintained in avian reservoirs (*1*). The segmented influenza A genome facilitates reassortment events in birds or intermediate hosts, such as swine and horses, leading to emergence of new variants potentially capable of causing zoonotic infections (*2*). Bats are major sources of zoonotic pathogens (*3*). In pioneering studies from 2012 and 2013, the first bat influenza A viruses, termed H17N10 and H18N11, were discovered in 2 bat species, *Sturnira lilium* (little yellow-shouldered bat) and *Artibeus planirostris* (flat-faced fruit-eating bat) (*4*,*5*).

Bat-associated influenza A viruses are phylogenetically highly divergent from avian-associated influenza A viruses in their hemagglutinin (HA) and neuraminidase (NA) genes, suggesting these viruses represent ancient influenza A strains (*2*). Consistent with their genetic divergence, bat-associated influenza A surface proteins lack typical hemagglutination and neuraminidase activities (*6*), leading to the terminology HA-like (HL) and neuraminidase-like (NL) for bat-associated influenza surface proteins.

So far, only 4 individual bat specimens yielded influenza A genomic sequences during the pivotal investigations (*4*,*5*). HL18NL11 has only been found in 1 *A. planirostris* bat captured in Peru in 2010 (*5*), challenging definite host assessments. To investigate bat influenza A virus epidemiology, we investigated bats in southern Brazil during 2010–2014. 

## The Study

For this study, we sampled 533 individual bats representing 26 species and 3 families across 28 sampling sites (Table 1). Bats were captured using mist nets, euthanized, and necropsied and were identified on the basis of morphological criteria by trained field biologists as described previously (*7*). Only intestine samples were available for virological analyses. The Instituto Brasileiro do Meio Ambiente e dos Recursos Naturais (21748–1), Instituto Ambiental do Paraná (235/10), and the ethics committee of the Institute of Biomedical Science from the University of São Paulo (56–18–03/2014) authorized sampling. 

**Table 1:** Bat species screened for influenza A virus, Brazil, 2010–2014*

Species	Family	No. samples	No. (%) PCR positive	Sampling site	Sampling years
*Artibeus fimbriatus*	Phyllostomidae	3	0	Iguaçu	2012
*Artibeus lituratus*	Phyllostomidae	129	2 (1.6)	**Iguaçu**, Central Paraná state, São Paulo cities	2010, 2011, **2012**, 2013, 2014
*Artibeus obscurus*	Phyllostomidae	1	0	São Paulo cities	2013
*Artibeus planirostris*	Phyllostomidae	4	0	Iguaçu, Central Paraná state, São Paulo cities	2010, 2012, 2014
*Carollia perspicillata*	Phyllostomidae	44	0	Iguaçu, Central Paraná state	2010–2012
*Cynomops planirostris*	Molossidae	6	0	São Paulo cities	2014
*Desmodus rotundus*	Phyllostomidae	15	0	São Paulo cities	2014
*Eptesicus furinalis*	Vespertilionidae	8	0	São Paulo cities	2013–2015
*Eumops auripendulus*	Molossidae	1	0	São Paulo cities	2014
*Eumops glaucinus*	Molossidae	44	0	São Paulo cities	2013–2015
*Eumops perotis*	Molossidae	8	0	São Paulo cities	2014–2015
*Glossophaga soricina*	Phyllostomidae	27	0	São Paulo cities	2013–2015
*Lasiurus cinereus*	Vespertilionidae	1	0	São Paulo cities	2013
*Lasiurus ega*	Vespertilionidae	1	0	São Paulo cities	2014
*Molossus molossus*	Molossidae	115	0	São Paulo cities	2013–2015
*Molossus rufus*	Molossidae	63	0	São Paulo cities	2013–2015
*Myotis nigricans*	Vespertilionidae	13	0	São Paulo cities	2013–2015
*Myotis riparius*	Vespertilionidae	1	0	São Paulo cities	2013
*Nyctinomops laticaudatus*	Molossidae	3	0	São Paulo cities	2014–2015
*Nyctinomops macrotis*	Molossidae	1	0	São Paulo cities	2014
*Phyllostomus discolor*	Phyllostomidae	2	0	São Paulo cities	2014
*Platyrrhinus lineatus*	Phyllostomidae	4	0	São Paulo cities	2014
*Promops nasutus*	Molossidae	1	0	São Paulo cities	2014
*Sturnira lilium*	Phyllostomidae	28	0	Iguaçu, Central Paraná state	2010–2012
*Tadarida brasiliensis*	Molossidae	9	0	São Paulo cities	2014
*Vampyressa pusila*	Phyllostomidae	1	0	Central Paraná state	2012
Total		533	2 (0.4)		


*Sampling sites were Parque Nacional do Iguaçu, Atlantic rainforest in western Paraná (Iguaçu); 26 cities across São Paulo state (São Paulo cities); and forest fragment in Paraná state (Central Paraná state). Bold indicates the site and year in which bats were captured that tested positive for influenza A virus.

**Figure 1:**
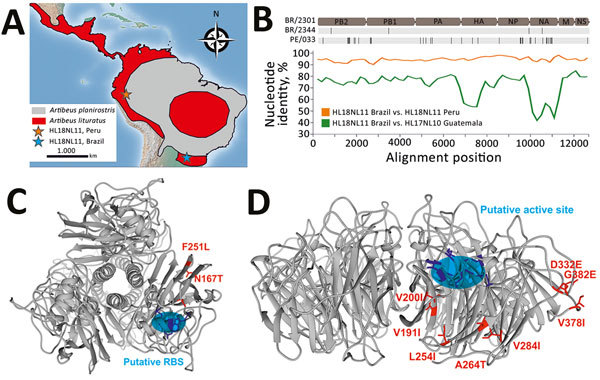
Bat influenza A(HL18NL11) virus detection and genomic characterization, Brazil, 2010–2014. A) Distribution of *Artibeus* species bats carrying HL18NL11 in Central and South America, according to the Red List of Threatened Species from the International Union for Conservation of Nature (https://www.iucnredlist.org). Orange star indicates the sampling site of an HL18NL11-positive bat in Peru (*5*); blue star indicates the sampling site of the HL18NL11-positive bats in Brazil for this study. Maps were created using QGIS2.14.3 (http://www.qgis.org) with data freely available from http://www.naturalearthdata.com. B) Top, schematic representation of the genome organization of A/great fruit-eating bat/Brazil/2301/2012 (HL18NL11) and amino acid exchanges (black lines) compared with A/great fruit-eating bat/Brazil/2344/2012 (HL18NL11) and Peru HL18NL11 (GenBank accession nos. CY125942–49). Nucleotide sequence identities between the concatenated HL18NL11 (Brazil), HL17NL10, and HL18NL11 (Guatemala and Peru) sequences were calculated in SSE version 1.2 (http://www.virus-evolution.org/Downloads/Software) with a sliding window of 200 and step size of 100 nt. C) Homology model of the HL protein of A/great fruit-eating bat/Brazil/2301/2012 viewed from the top, modeled on the published crystal structure retrieved from the SWISS-MODEL repository (https://www.swissmodel.expasy.org). The putative RBS is shown in blue, 3 highly conserved residues (W153, H183, and Y195) in HAs and HLs are in purple, and amino acid substitutions between Brazil strains and the Peru prototype strain are in red. D) Homology model of the NL of A/great fruit-eating bat/Brazil/2301/2012 viewed from the top, constructed as in panel C. The putative active site is shown in a blue circle, the 6 residues (R118, W178, S179, R224, E276 and E425) conserved in influenza A virus neuraminidase genes are in purple, and amino acid substitutions between Brazil strains and the Peru prototype strain are in red. HA, hemagglutinin; HL, HA-like; NL, neuraminidase-like; RBS, receptor-binding site.

We tested intestine specimens from all bats using 2 highly sensitive, broadly reactive nested reverse transcription PCRs targeting different regions of the influenza A polymerase basic (PB) 1 gene (*5*,*8*). Positive results on both tests came from only 2 samples, from *Artibeus lituratus* great fruit-eating bats captured on March 7 and March 12, 2012, at 2 locations separated by 12 km in an Atlantic rainforest patch. No other sample was positive, yielding a 10.0% (2/20) overall detection rate in this site and 16.7% (2/12) detection in *A. lituratus* bats from this site (Table 1; Figure 1, panel A). Neither bat testing positive for influenza A virus showed signs of disease. *A. lituratus* bats were the most abundantly sampled species (Table 1). 

The low overall influenza virus detection rate in this study (0.4%, 95% CI 0.0%–1.5%) was not significantly different by Fisher exact test from the previous 2 studies (1/110 bats for HL18NL11 [0.9%, 95% CI 0.0%–5.5%; p = 0.43]; 3/316 bats for HL17NL10 [1.0%, 95% CI 0.0%–2.9%; p = 0.37]). Apparently low rates of acute influenza A virus infection in bats are not consistent with high seroprevalence of 72% in different bat species according to a preliminary investigation (*5*) and may hint at seasonal variation in bat influenza virus infections, comparable to other batborne RNA viruses (*9*).

Sanger sequencing of the screening PCR amplicons suggested close genetic relatedness of the strains circulating in Brazil with the HL18NL11 strain circulating in Peru. Virus concentrations in the positive intestine specimens as determined by strain-specific quantitative real-time reverse transcription RT-PCR (Appendix Table 1) were high (1.5 × 10^9^ and 4.9 × 10^10^ RNA copies/g of tissue). High HL18NL11 concentrations in intestinal specimens are consistent with qualitative data from the pioneering study on HL18NL11 (*5*) and may suggest intestinal tropism and potential fecal shedding into the environment.

We determined the full coding sequence of all 8 segments of the viral genomes using primers aiming at amplifying overlapping regions of bat influenza A virus genomes (GenBank accession nos. MH682200–15) (Appendix Table 1). The 2 HL18NL11 variants in Brazil differed by 15 nt from each other across the combined 8 genomic segments. Four of those substitutions were nonsynonymous, causing amino acid exchanges in the PB2 (V203I), PB1 (R334K), nucleoprotein (G484S), and NA (V191I) genes (Table 2; Figure 1, panel B). This finding suggests recent common ancestry of the HL18NL11 variants identified in the 2 positive bats and was consistent with their detection in the same site 5 days apart. Comparison of the full coding sequence of the novel HL18NL11 variants revealed high sequence identity between the Peru and the Brazil strains, 93.5%–96.9% nucleotide identity across all 8 genomic segments (Table 2). The genomic relatedness of Peru and Brazil HL18NL11 strains was surprising given a time span of 2 years, a geographic distance exceeding 2,000 km, and 2 different bat species that tested positive in our study and the previous study (*5*).

**Table 2:** Comparison of influenza A(HL18NL11) strain found in bats in Brazil with prototype strains from Peru

Gene	Nucleotide sequence identity	Amino acid exchange site	
		A/great fruit-eating bat/Brazil/2301/2012 (HL18NL11a)	A/great fruit-eating bat/Brazil/2344/2012 (HL18NL11b)
PB2	93.6%	V76I, R471K, T473N, V478I, I559V, R574K, S631N	V76I, **V203I**, R471K, T473N, V478I, I559V, R574K, S631N
PB1	93.7%	V54I, T56V	V54I, T56V, **R334K**
PA	94.4%	T70A, R116K, D158N, V231I, T254S, I552V, R711G	T70A, R116K, D158N, V231I, T254S, I552V, R711G
HL	96.0%	N167T, F251L	N167T, F251L
NP	96.8%–96.9%	N20T, K350R, L357M, I380L, I387V	N20T, K350R, L357M, I380L, I387V, **G484S**
NL	93.5%	I11V, I15L, V82I, V200I, L254I, A264T, V284I, D332E, V378I, G382E	I11V, I15L, V82I, **V191I**, V200I, L254I, A264T, V284I, D332E, V378I, G382E
M	95.4%	None	None
NS1	94.4%	R57K	R57K
*Bold indicates amino acid exchanges occurring in only 1 of the 2 Brazil strains compared to the Peru prototype strain. HA, hemagglutinin; HL, HA-like; M, matrix; NA, neuraminidase; NL, neuraminidase-like; NS, nonstructural protein; NP, nucleoprotein; PA, polymerase acidic; PB, polymerase basic.			

All critical amino acid residues associated with influenza A virus replication and entry (*4*,*5*) were conserved between the Brazil and the Peru HL18NL11 strains, including the HA monobasic cleavage site motif PIKETR/GLF (*5*). Thermodynamic modeling revealed that the amino acid exchanges observed between the Brazil and Peru HL18NL11 strains did not alter the tridimensional structure of the HL and NL proteins, and neither mapped to the putative receptor binding site of the HL protein (Figure 1, panel C), nor to the putative active site of the NL protein (Figure 1, panel D) (*6*). This result suggests preservation of the biologic activity of these glycoproteins in different bat species and supported a broadly conserved cellular receptor of bat influenza A viruses that differs from sialic acid receptors used by avian-associated influenza A viruses (*10*). Significantly fewer amino acid exchanges were observed between the HL proteins of Brazil and Peru bat influenza virus than between the respective NL proteins (p = 0.007 by Fisher exact test) (Table 2). The apparently low rate of nonsynonymous substitutions in the HL-encoding genes of bat influenza A virus variants was reminiscent of strong purifying selection acting on the hemagglutinin genes in avian-specific influenza A virus strains (*11*). This finding may suggest comparable evolutionary dynamics between chiropteran and avian reservoirs. Definite assessments will require considerably larger datasets of bat influenza A virus strains.

**Figure 2:**
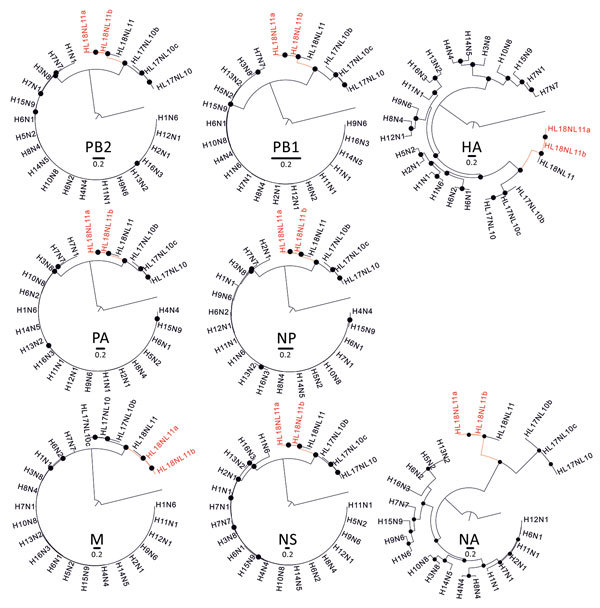
Phylogenetic relationships between bat influenza A viruses from Brazil and reference viruses. Phylogenetic trees show comparison of the 8 segments of representative influenza A virus genomes (PB2, PB1, PA, HA/HL, NP, NA/NL, M, NS) with A/great fruit-eating bat/Brazil/2301/2012 (HL18NL11a; GenBank accession nos. MH682200–7) and A/great fruit-eating bat/Brazil/2344/2012 (HL18NL11b; GenBank accession nos. MH682208–15), shown in red. Maximum-likelihood trees were inferred using a general time-reversible substitution model with a gamma distribution and invariant sites. Black dots represent bootstrap values >75% (1,000 replicates). Trees were generally rooted using influenza B/Lee/1940 (GenBank accession nos. DQ792894–901) (data not shown). Trees were constructed by using MEGA 6.0 (http://www.megasoftware.net). HA, hemagglutinin; M, matrix; NA, neuraminidase; NS1, nonstructural protein 1; NP, nucleoprotein; PA, polymerase acidic; PB, polymerase basic. Scale bars indicate nucleotide substitutions per site.

*A. lituratus* bats and *A. planirostris* bats, in which HL18NL11 was originally detected in Peru, represent closely related, yet genetically and morphologically clearly distinct bat species (*12*). The distribution of these bat species overlaps (Figure 1, panel A), potentially facilitating virus exchange across the populations. Phylogenetic analyses confirmed the close genetic relationship between Peru and Brazil HL18NL11 variants across all 8 segments (Figure 2; Appendix Table 2), suggesting lack of reassortment events according to the available data. Our data thus suggest host associations of HL18NL11 beyond the species level, comparable to genus-level host associations of other batborne RNA viruses such as coronaviruses (*13*).

## Conclusions

The zoonotic potential of HL18NL11 is unclear, yet human-derived cell lines were susceptible to infection by chimeric vesicular stomatitis virus pseudotyped with HL18 (*14*). The abundance of *A. lituratus* bats within Latin America (Figure 1, panel A) may thus facilitate spillover infections into other vertebrates across an underrecognized geographic and host range. Finally, *Artibeus* spp. bats have been used previously for infection studies including viruses with evolutionary origins in bats, such as Middle East respiratory syndrome coronavirus (*15*). The relatively large body size of *A. lituratus* bats (≈65 g) and ease of keeping these bats under laboratory conditions may thus facilitate experimental infection studies for HL18NL11 to elucidate the exact sites of HL18NL11 replication, receptor usage, and mode of transmission.

Appendix Additional information related to bat influenza A(HL18NL11) virus in Brazil, 2010–2014.
