# Supplementary material for: Bat Influenza A(HL18NL11) Virus in Fruit Bats, Brazil
Source: Emerg Infect Dis. 2019 Feb;25(2):333–7. doi: 10.3201/eid2502.181246 (PMC6346480; doi:10.3201/eid2502.181246)
Supplement: Appendix — Additional information related to bat influenza A(HL18NL11) virus in Brazil, 2010–2014. [file 18-1246-Techapp-s1.pdf]

Article DOI: https://doi.org/10.3201/eid2502.181246

Bat Influenza A(HL18NL11) Virus in Fruit Bats, Brazil

Appendix

RNA was extracted from 30 mg of tissue using the RNeasy Kit (QIAGEN, www.qiagen.com), followed by random hexamer-driven cDNA generation using the Superscript III reverse transcription kit (Thermo Scientific, www.thermofisher.com). Reactions were set up in a final volume of 20 µL with 10 µL of total RNA, 0.6 µM of primers, 1x First-Strand Buffer, 0.5 mM (each) dNTP, 3.3 mM DTT, 1 µg BSA, 40 U of Rnase OUT and 200 U SuperScript III. Hemi-Nested PCRs were performed in 25 µL reactions with 1 µL of cDNA (for first rounds) or PCR template (for second rounds), 1 µM of each primer, 2.0 mM $MgCl_2$, 0.2 mM (each) dNTP and 1 U Platinum Taq Polymerase (Thermo Scientific). Thermocycling included a touchdown protocol with 94°C/3 min, 94°C/15 s, 68°C/30 s (–1°C per cycle) and 72°C/1 min during the first 10 cycles, followed by 45 cycles of 94°C/15 s, 58°C/30 s, 72°C/90 s, and 72°C for 7 min. One-step real-time RT-PCR–based quantification was done using primers targeting the PB1 gene and performed in 25 µL reaction volumes with 5 µL of RNA, 2.0 mM $MgCl_2$, 0.2 mM (each) dNTP, 0.4 µM of each primer, 0.3 µM of probe, and 1x PCR buffer with OneStep SSIII/Taq Enzyme Mix (Thermo Scientific). Amplification involved 55°C for 20 min (RT), followed by 94°C/3 min and 45 cycles of 94°C/15 s and 58°C/30 s with fluorescence read at the 58°C step, cooling at 40°C for 30 s on a LightCycler 480 thermocycler (Roche, www.roche.com). Quantification relied on photometrically quantified cRNA transcribed using the Megascript kit (Asuragen, www.asuragen.com) from a pCR4 vector containing the PCR target region (Thermo Scientific).

Appendix Table 1. Primers used for genomic amplification and sequencing

Gene	Primer name and position	Sequence 5′- 3′
PB2	BatFluPB2F1	AGCAGAAGCAGGTCARAGATTG
	BatFluPB2F2–632	TGGTTGCATACATGCTKGAAAGG
	BatFluPB2F3–1333	AGRCATTTCCAAAARGACTC
	BatFluPB2F4–1542	AAATGAAAAGGGAGAAYTWCT
	BatFluPB2F5–1336	CATTTCCAAAAAGACTCTGGAG
	BatFluPB2F6–677	GGTTCCTGCCAGTTGCAGG
	BatFluPB2F7–727	CACCTAACCCAAGGCACGTG
	BatFluPB2F8–2089	GAGTCAGCAGTGTTAAGAGG
	BatFluPB2F9–2160	CGCAGAGCTGGATAAATTAGG
	BatFluPB2R7–763	ATTCTCWGCTTCMCCTCCTGG
	BatFluPB2R8–746	CCTGGWGTRTAYTGTTGCTCCC

Gene	Primer name and position	Sequence 5′- 3′
	BatFluPB2R9–1678	TCCCAGTTYTTTAGTATCCAGTG
	BatFluPB2R11–1483	ATTCTYTCGTTGAAAGAGTATTCATC
	BatFluPB2R10–1762	CCYTTGGGKATTAAAGTTTGAAAWGG
	BatFluPB2R12–2256	GCTGTCTGGCTATCAGTAAGT
	BatFluPB2R13–1426	CCGGTAGTATTCCCATAATTCC
	BatFluPB2R14–1474	GACTTATTCTTATCCCAACCAGTG
	BatFluPB2R1–2314	AGTAGAAACAAGGTCATTTTTAGTG
	BRbatFluPB2F10–1000	GCTTTGGAGGCTATAACTTTAAG
	BRbatFluPB2F11–566	GATGCACAATTAGCGATCACC
	BRbatFluPB2F12–627	CCAATTATGGTTGCATACATGC
	BRbatFluPB2R15–1572	TGCTTCACTTACTTCTTCTGG
	BRbatFluPB2R16–1611	GGGATGAATTGTAGTTTATTGG
	BRbatFluPB2R17–1066	GTTAATGTTTGAAGGTTTCCAG
PB1	BatFluPB1F1	AGCAGAAGCAGGCAAACTATT
	BatFluPB1F2–1242	AGGRATGATGATGGGVATGTTC
	BatFluPB1F3–711	AARGAYGCAGAGAGAGGWAAA
	BatFluPB1F4–1593	ATGAGYATAGGMACAACAGT
	BatFluPB1F5–1923	CACCATATGGARGTIGAAAGCAC
	BatFluPB1F6–1625	CATGATMAACAATGATCTAG
	BatFluPB1F7–1251	ATGGGSATGTTCAATATGC
	BatFluPB1F8–1594	TGAGTATAGGMACAACAG
	BatFluPB1F12–1926	CCATATGGAAGTCGAAAGCAC
	BatFluPB1F13–1958	TAATAATGCCAGCCCACG
	BatFluPB1F14–1961	TAATGCCAGCCCACGGGCCA
	BatFluPB1R3–759	CAAAYCCTCTDATTTGCATTCC
	BatFluPB1R4–1007	TGAACCATTCMGGTTGICCT
	BatFluPB1R5–2012	GGTGTCCATGAATGAGTTG
	BatFluPB1R6–1007	TGAACCATTCMGGTTGICCT
	BatFluR1PB1–2322M2M1–1010	AGTAGAAACAAGGCATTT
	BRbatFluPB1F10–1574	GAAACAATGAGTCTGCTGA
	BRbatFluPB1F11–1594	ATGAGTATAGGCACAACAG
	BRbatFluPB1R5–1424	CTACCAATTTGCAGATTCTATAG
	BRbatFluPB1R7–1076	CATGTATCCCCGTCCAAGTC
PA	BatFluPAF1	AGCAGAAGCAGGTACTTARAC
	BatFluPAF2–680	CCWCCATCATTCAAGGACTAT
	BatFluPAF3–1144	GGAYTTTGAAGATTGTAAAG
	BatFluPAF4–1266	CTCAAACTGGATYGAATTTGATG
	BatFluPAF6–1916	TGCAGAGTTCTTCTAGC
	BatFluPAF7–1943	TTCAACAGCATATATGCT
	BatFluPAF8	GCAGGTACTTARACAATGGAGAA
	BatFluPAR7–1176	GGWTCACTTTTGTATTGGAACA
	BatFluPAR8–1738	CATCTTCTCATTTCCATTCCC
	BatFluPAR9–1457	GTCTCTACATTTRGTRATTATTGG
	BatFluPAR10–1438	TGGKATYACYTGATATTCCTCCA
	BatFluPAR11–2089	GACCCAAGGATCATTAATG
	BatFluPAR12–273	CAATAGTCCAGGCAACATTTC
	BatFluPAR13–300	CTATATTGGTCATGTTGCATATTG
	BatFluPAR1–2198	AGTAGAAACAAGGTACTTT
HL	FluHaF1	AGCAGAAGCAGGGTSAYTATTAYTC
	BatFluHaF2–946	ACAGYACMCTGCCYTTTCA
	BatFluHaF3–991	AYTGTCCYAAATATGTGAARGC
	BatFluHaF4–756	GAGTTGTCAATCCTAATCAGAATC
	BatFluHaF5–951	CATCAAAATGCGATTGGAGATTGC
	BatFluHaF6–1247	AACTGCCAAAGAATTCAAC
	BatFluHaR4–1305	CCATCATCAACTCTGTCACTGAG
	BatFluHaR5–1104	CATCAATCAWYCCTTGCCATCC
	BatFluHaR6–1729	GATTGACATTAGCTAACAC
	BatFluHaR7–242	GTTTCCCATAAGCCATGCAGG
	BatFluHaR1–1775	AGTAGAAACAAGGGTSTTT
NP	BatFluNPF1	AGCAGAAGCAGGGTTAATAATC
	BatFluNPF2–24	CACATTGTGACATTTAAAGATG
	BatFluNPF3–739	CCACAGAAAGCAATGGTTGA
	BatFluNPF4–511	AATGGACCCAAGRATGTGCTC
	BatFluNPF5–1349	ACTGACATGAGAACTGA
	BatFluNPF6–1386	ATTCTGATCCCAAAGAC
	BatFluNPF7–1344	GAATCACTGACATGAGAACTG
	BatFluNPF8–1303	GAAAACAATAATGGCTGCA

Gene	Primer name and position	Sequence 5′- 3′
	BatFluR1NP-1541NEP-878	AGTAGAAACAAGGGTATT
	BatFluNPR5–1189	GTCTTGATTGCCCAATAATG
	BatFluNPR6–1482	GAAATAAGAACCCTCGTCATTC
	BatFluNPR7–251	GGTATTTGTTCCTTCTTTCGTC
NL	BatFluNaF1	AGCAGAAGCAGGAGTTTTTMA
	BatFluNaF2–892	CAAATCTYTGGAATGATGCCAA
	BatFluNaF3–900	TGGAATGATGCCAARAGRCC
	BatFluNaHL18F-623	TGTGAGCATCCTTTATGGAG
	BatFluNaHL17F-629	AACAGACACTTTCTCGGCCAGCA
	BatFluNaF8–1232	TTATGAATCACGTGATTG
	BatFluNaR8–310	ATGCTGCACAGATTCTCTC
	BRbatFluNaR8–231	TGTTGAACTGTAGAAGCT
	BRbatFluNaR9–362	GTCAGCATTTGTTCATCA
	BRbatFluNaR10–993	ATTTGTAGTGTCAATTCC
	BatFluNaR12–1396	CAAGGAGTTTTTTCTTATACATC
	BatFluNaR13–1395	CAAGGAGTTTTTTCTTATACATCC
	NewBatFluNaF1	AGCAGAAGCAGGAGT
	newBatFluNaF5–497	CAGTGTAAAGTTAGGAGACC
	newBatFluNaF6–932	CCAGTCCTTCACTTACAC
	newBatFluNaF7–949	CTTTCAAGGAGCCATGCTTG
	newBatFluNaR4–511	CAGGTGTTGGAGGGTCTC
	newBatFluNaR5–573	CACTGAAAGCCATCATG
	newBatFluNaR6–1101	TCCTTTCTTGGATCCTGG
	newBatFluNaR7–1044	ATCATGAAACCTTGGATTCC
	inselnBatFluNaF1–177	AGCTGTCCAAACGGGACTTCTG
	inselnBatFluNaF2–820	GGAACATYTSCHGGCTGGAAG
	inselnBatFluNaF3–916	ACAAATCTYTGGAATGATGCCAA
	inselnBatFluNaR1–1129	CAAATCCWTTCTKGGATCCTGG
	inselnBatFluNaR2–655	CWGTTATWATTYCTCCATAWAGGAT
	inselnBatFluNaR3–596	GACAGTCCACTGAAAGCCATC
	BRbatFluNaF9–541	TTGAAGCTGTTGGCTGGA
	BRbatFluNaF10–598	TGTCCGTTGCAGGAGACG
	BRbatFluNaF13–1020	AAGACAACAACAGAGGGAGA
	BRbatFluNaR8–231	TGTTGAACTGTAGAAGCT
	BRbatFluNaR9–362	GTCAGCATTTGTTCATCA
	BRbatFluNaR10–993	ATTTGTAGTGTCAATTCC
	BatFluNaR12–1396	CAAGGAGTTTTTTCTTATACATC
	BatFluNaR13–1395	CAAGGAGTTTTTTCTTATACATCC
M2/M1	BatFluM2M1F1	AGCARAAGCAGGCATTATYCAAA
	New BatFluM2M1F1	AGCARAAGCAGGCATTATYC
	BatFluM2M1F2–575	CACTGCHAARGCCATGGARCAA
	BatFluM2M1F3–621	GCTGAAGCAATGGAAATTGC
	BRbatFluM2F4–547	GACATGAAAACCGAATGGCAAC
	BRbatFluM2F5–631	TGGAAATTGCTTCACAAG
	BatFluM2M1R2–741	ACCAGAARAGRATGGGAAT
	BatFluM2M1R3–687	CACCCAACAACTTCCAGTGG
	BatFluM2M1R4–752	CTGCATCTGGATTCCCATC
	BatFluM2M1R5–690	GGCCACTGGAAGTTGTTGG
NEP/NS1	BatFluNEPF1	AGCAGAAGCAGGGTATCTAAAG
	New BatFluNEPF1a	AGCAGAAGCAGGGTATCTAA
	BatFluNEPF2–16	TCTAAAGACATAATGGAAYC
	BatFluNEPF2–40	CCGACAACTATCGCATTTCAG
	BatFluNEPF3–514	AACCCTCTGTCTTTTGTTACAG
	BatFluNEPF4–529	GTTACAGGACATACTGGAGAG
	BatFluNEPR2–583	GGATTTGAATGGAATGATAAC
	BRbatFluNEPR3–241	CATAGTAAGGCATGGCATC
	BRbatFluNEPR4–327	GATCATAATCCAATTTCTG
PB1 quantitative real-time RT-PCR	FluBR-rtF1	TGCAGAAGAACTGAAYACTATAAGCTT
	FluBR-rtR	TGAACATSCCCATCATCATTCC
	Probe FluBR-rtP	FAM-TYGATGGGACAGCRTCACTGAGCCC-BHQ1

*HL, hemagglutinin-like; M2/M1, matrix protein 2 and matrix protein 1; NL, neuraminidase-like; NP = nucleocapsid, NEP/NS1, nuclearexport protein and non-structural protein 1; PA, PB1, PB2, polymerase genes.
†Numbers in primer names indicate the first nucleotide targeted in the Peruvian HL18NL11 prototype strain. For the degenerated bases, R = G/A, Y = C/T, S = G/C, W = A/T, M = A/C, K = G/T, H = A/C/T, I = inosine. FAM, 6-carboxyfluorescein; BHQ1, Black Hole Quencher1.

Appendix Table 2. Representative viruses used in phylogenetic analysis of Brazilian bat influenza A (HL18NL11) virus

NA	NA	Collection date	PB2 gene	PB1 gene	PA gene	HA gene	NP gene	NA gene	M gene	NS gene
H1	N1	1978	CY020300	CY020299	CY020298	CY020293	CY020296	CY020295	CY020294	CY020297
H1	N6	1977 Aug 2	CY004465	CY004464	CY004463	CY004458	CY004461	CY004460	CY004459	CY004462
H2	N1	1990 Apr 18	CY005420	CY005419	CY005418	CY005413	CY005416	CY005415	CY005414	CY005417
H3	N8	1963	CY032300	CY032299	CY032298	CY032293	CY032296	CY032295	CY032294	CY032297
H3	N5	1999 Oct 7	CY060258	CY060259	CY060260	CY060261	CY060262	CY060263	CY060264	CY060265
H4	N4	1979	CY045270	CY045269	CY045268	CY045263	CY045266	CY045265	CY045264	CY045267
H5	N2	1984 Feb 9	CY005764	CY005763	CY005762	CY014640	CY005760	CY014641	CY005759	CY005761
H6	N1	1979 Jan 1	CY005671	CY005670	CY005669	CY014623	CY005667	CY014624	CY005666	CY005668
H6	N2	2004 Dec 5	CY045478	CY045477	CY045476	CY045471	CY045474	CY045473	CY045472	CY045475
H7	N1	1934	CY077417	CY077418	CY077419	CY077420	CY077421	CY077422	CY077423	CY077424
H7	N7	1977	CY036902	CY036901	CY036900	CY036895	CY036898	CY036897	CY036896	CY036899
H8	N4	1968	CY005831	CY014662	CY005830	CY014659	CY005829	CY014660	CY005828	CY014661
H9	N6	1988 May 17	CY004574	CY004573	CY004572	CY005934	CY004570	CY004569	CY004568	CY004571
H10	N8	1965	CY005800	CY005799	CY014645	CY014644	CY005797	CY005796	CY005795	CY005798
H11	N1	1986 Nov 6	CY017772	CY017771	CY017770	CY017765	CY017768	CY017767	CY017766	CY017769
H12	N1	1983 Aug 6	CY005350	CY005349	CY005348	CY006006	CY005346	CY005345	CY005344	CY005347
H13	N2	1986 Jun 1	CY003901	CY003900	CY003899	CY005914	CY003897	CY003896	CY003895	CY003898
H14	N5	1982	CY130101	CY130100	CY130099	CY130094	CY130097	CY130096	CY130095	CY130098
H15	N9	1983	CY005724	CY005723	CY005722	CY006033	CY005720	CY005719	CY005718	CY005721
H16	N3	1988 May 16	CY004567	CY004566	CY004565	CY005933	CY004563	CY014569	CY004562	CY004564
H17	N10	May 2009	CY103873	CY103874	CY103875	CY103876	CY103877	CY103878	CY103879	CY103880
H17	N10	May 2009	CY103881	CY103882	CY103883	CY103884	CY103885	CY103886	CY103887	CY103888
H17	N10	Sep 2010	CY103889	CY103890	CY103891	CY103892	CY103893	CY103894	CY103895	CY103896
H18	N11	2010	CY125942	CY125943	CY125944	CY125945	CY125946	CY125947	CY125948	CY125949
H18	N11	2012 Mar 7	MH682200	MH682201	MH682202	MH682203	MH682204	MH682205	MH682206	MH682207
H18	N11	2012 Mar 12	MH682208	MH682209	MH682210	MH682211	MH682212	MH682213	MH682214	MH682215

*The influenza B strain used as an outgroup was B/Lee/1940 (accession numbers DQ792894–901).
